# Livestock feed resources used as alternatives during feed shortages and their impact on the environment and ruminant performance in West Africa: a systematic review

**DOI:** 10.3389/fvets.2024.1352235

**Published:** 2024-05-24

**Authors:** Nouroudine Alimi, Alassan S. Assani, Hilaire Sanni Worogo, Nasser Mohamed Baco, Ibrahim Alkoiret Traoré

**Affiliations:** ^1^Laboratoire d’Ecologie, de Santé de Production Animales (LESPA), Faculté d’Agronomie (FA), Université de Parakou (UP), Parakou, Benin; ^2^Laboratoire Société-Environnement (LaSen), Faculté d’Agronomie (FA), Université de Parakou (UP), Parakou, Benin

**Keywords:** nutritional value, environmental impact, ruminants, alternative feeds, West Africa

## Abstract

Ruminant feed is a major problem for the livestock sector in West African developing countries causing animal nutritional diseases, reducing ruminant production, and *creating a massive ecological crisis through greenhouse gas emissions*. Alternative feeds, which include agro-industrial by-products, fodder trees, crop residues, insects, fodder legumes, algae, and pulses, constitute enormous feed resources for livestock in Africa. This study was conducted in accordance with the methodological recommendations of PRISMA (Preferred Reporting Items for Systematic Reviews and Meta-Analyses). We conducted a literature search using Google Scholar, Web of Science, and Scopus to identify documents related to alternative ruminant feeds using the following keywords: alternative feeds, ruminant products, environmental impacts, and West Africa. Those that met the inclusion criteria were included, resulting in 44 articles published between 2013 and 2023. These studies included 45 alternative feeds divided into six groups, including agro-industrial by-products (48.89%), followed by fodder trees (17.78%), crop residues (13.33%), insects (8.89%), fodder legumes (6.67%) and seaweeds (4.44%). Our results revealed that alternative feed resources and their effects on ruminant’s performances and environment are poorly known in West Africa, which limits their inclusion in rations and sometimes leads to their misuse. Future research should focus on these aspects in order to make efficient use of these resources to improve ruminant milk and meat production.

## 1 Introduction

In the face of population growth and land use through urbanization, which absorbs arable land and therefore the availability of animal feed resources, livestock production in Africa is no longer able to meet the population’s demand for meat and dairy products. It is facing a huge feeding problem (1), which is a major cause of low milk and meat production in West Africa (2). This situation can be explained by the nutritional characteristics of grown forage (3) and a lack of knowledge on rationing techniques, resulting in lower animal productivity and considerable greenhouse gas emissions (4), contributing to global warming (5). It is well known that emissions cause an increase in temperature, and a decrease in rainfall (6) and have a significant impact on pastoral resources. This situation reduces the conditions necessary for ruminant production and makes West Africa the most affected by the effects of climate change in the world (7). The livestock sector contributes to global N flows through the application of synthetic N fertilizers and manure to both croplands and grasslands, the management and accumulation of manure, and the transport of N-rich products such as feed, food, and manure (8). In some cases, excess nitrogen is a source of air pollution (9). To ensure food security with more livestock production, it is necessary to use alternatives in feeding practices. Thus, alternative feeds appear to be a promising option to improve the feed supply chain for ruminants and strengthen their availability in dry and rainy seasons. Alternative feeds also help to improve milk and meat production at a lower cost. Rice straw and other crop residues such as cereal stalks are rich in cellulose and provide nutrients for the growth of rumen microbes (10). Crop residues and agro-industrial by-products used as alternative feeds also deserve to be preserved for the future of ruminant farming. Furthermore, Idrissou et al. (11) found that Djallonké sheep supplemented with *Leucaena leucocephala* and *Gliricidia sepium*, respectively, had significantly higher average daily gains in Benin. Studies on the effects of cassava and banana peels (12) showed that incorporating dried banana peels at 20% in the feed accelerated sheep growth and average daily weight gain. Moreover Kiéma et al. (13) showed that the use of *Faidherbia albida* pods increase average daily weight gain of young bulls therefore seems to be an alternative way of improving livestock production (14). According to Pamo et al. (15) who carried out studies on the chemical composition and effect of supplementation with *Calliandra calothyrsus* and *Leucaena leucocephala* on milk production in goats, fresh leaves of *Calliandra calothyrsus* and *Leucaena leucocephala* doubled the production (361 g/d) in dairy goats. A study by Sanogo et al. (16) on the effect of cereal straw, cowpea fodder, and cotton cake on improving milk production found that the average cumulative milk production was 140 liters for cows in stalls. Furthermore, the results of Gbenou et al. (17) revealed that the production of cows (Gir × Borgou) with the supplementation of 2 kg of sorghum meal resulted in a production of 3.3 kg of milk/day. According to Ahmed et al. (18) the combination of micro- and macroalgae as ruminant feeds can replace the expensive conventional sources in animal diets and help to reduce feeding costs and livestock impacts on environmental. Biologically active compounds, such as flavonoids found in by-products of the winery industry and citrus fruits, are gaining attention for their ability to modulate the immune system of ruminants (19), due to their positive effects on milk quantity and quality (20). According to Jalal et al. (21), *fruit and vegetable by-products can be used as an alternative feed source for sustainable ruminant nutrition and production*. The use of alternative feeds in ruminant nutrition would provide benefits and help alleviate the environmental problems associated with waste disposal (22). Including these by-products in ruminant diets could reduce the environmental impact of their disposal (23) and promote the growth of a circular economy by recycling the biomass derived from crop production (24). From a socio-economic point of view, it is possible to reduce ration costs, improve the income of livestock farmers, and develop the market for livestock feeds. Although these feeds are well known in West Africa, it has been observed that huge quantities are wasted. It is therefore necessary to focus on the availability, nutritional value, technical and economic limitations of these feeds, which vary from one agro-ecological zone to another, and consequently from one country to another. This review summarizes the results of various studies carried out on the nutritional value, ruminant products and environmental impact of using alternative feeds. The aim of this review was to enhance the value of locally available alternative feeds for ruminant feeding in all seasons through their efficient use in the formulation of balanced, climate-sensitive rations to improve household incomes and protect the environment.

## 2 Methods

**Figure 1:**
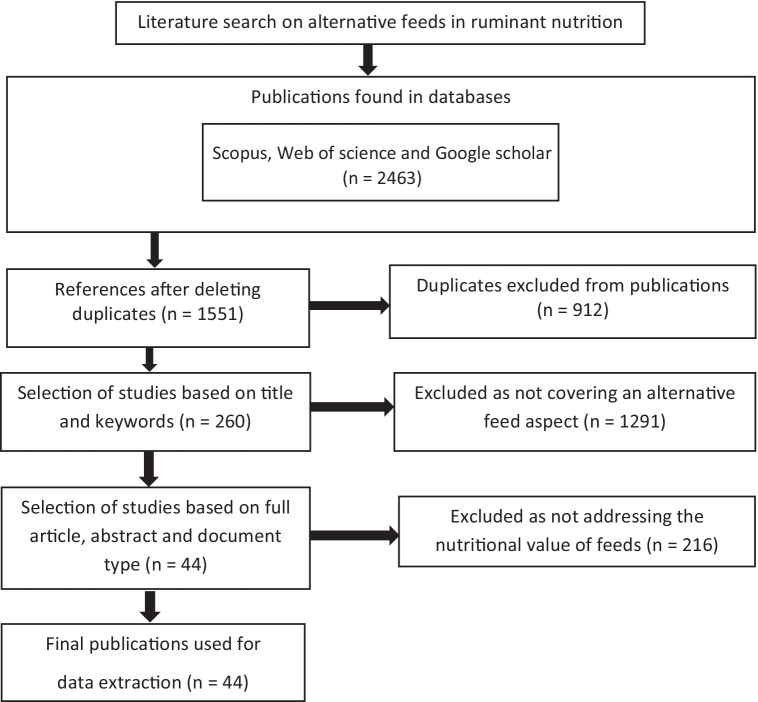
Document selection diagram.

Reporting Systematic Reviews and Meta-Analyses of Studies were used for the current study. Recent studies conducted principally in West Africa (Benin, Burkina Faso, Nigeria, Niger, Mali, and Côte d’Ivoire) from 2013 to 2023 were accessed through Google Scholar, Web of science, and Scopus. The downloaded documents were retrieved using a combination of the following keywords: alternative feeds, ruminant products, and environmental impacts. The keywords were used first in French and then in English in order to obtain as many publications as possible. The references found were imported into Zotero software version 5.0.94. Documents were selected based on the recommendations of (25, 26) by using a two-step process based on predefined criteria (Figure 1). The selection was based on titles, abstracts, and keywords. In total, 2,463 articles were obtained after excluding duplicates. In the second step, we based the selection on publication date, language (English or French), and research subject (alternative feeds for ruminants) in West Africa. As soon as the study topic appeared in any of the documents or was addressed in any other way, the article was downloaded and thoroughly checked. The selection criteria set out above were applied to the initial articles, resulting in the selection of 44 scientific publications (Figure 1). Publications for this synthesis were not analyzed as the only ones on the subject but were intended to provide a representative overview of alternative ruminant feeds in West Africa. The 44 articles finally selected were all in English.

### 2.1 Data management, review, and statistical analysis

Selected documents were examined in detail and underwent a complete review. The final publications were imported into Zotero, which identified the nature or type of document that was being recorded. Questions were then developed following the method by (27) method to show how alternative feeds are used in West Africa based on literature published between 2013 and 2023. We took into account general characteristics of the articles such as title, year of publication, country of study, affiliation of the main author, and links to alternative feeds.

## 3 Results and discussion

### 3.1 Number and type of documents

**Figure 2:**
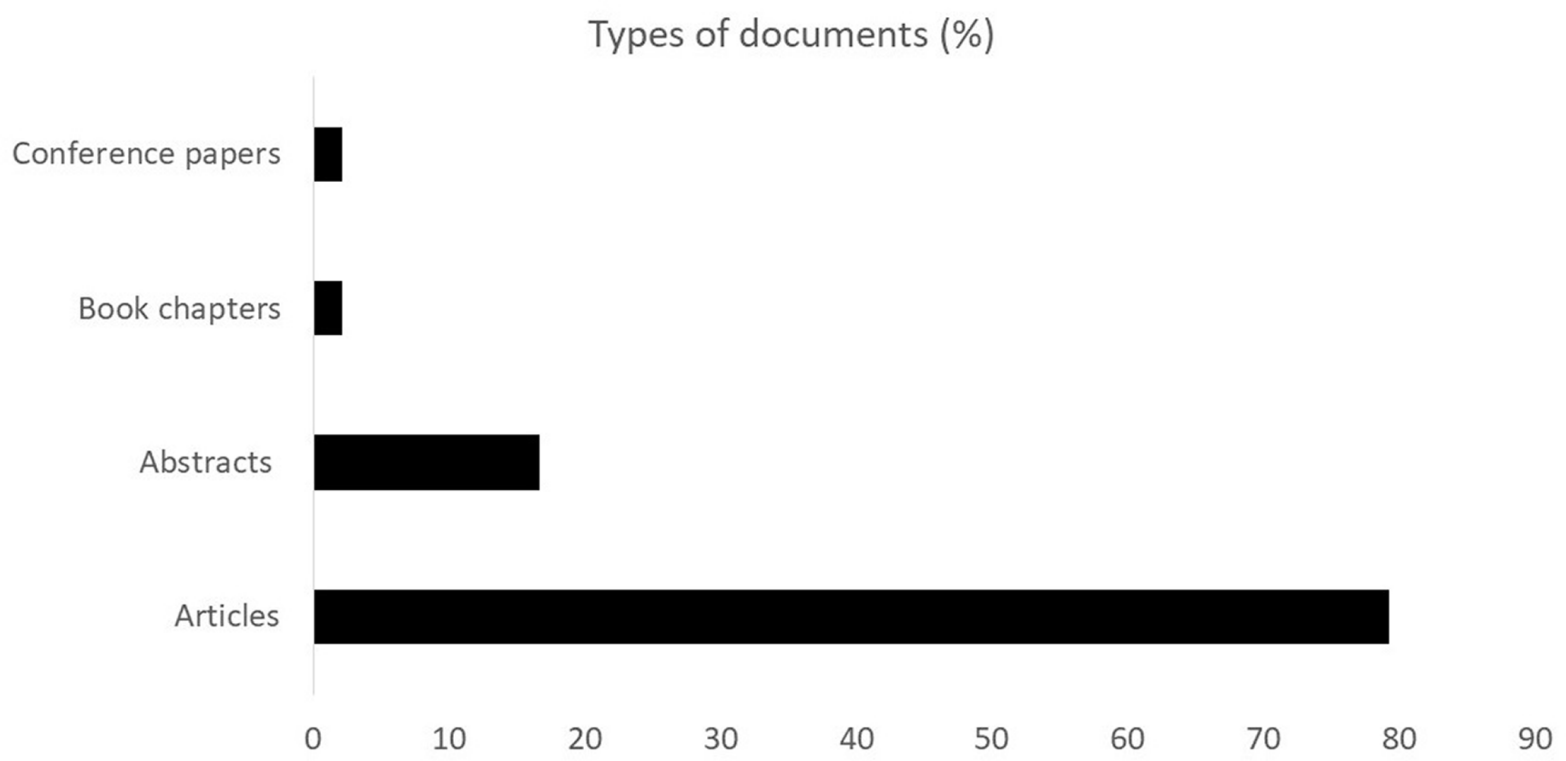
Percentage occurrence of document types.

The documents found include articles (79.2%), which are the most prevalent documents, followed by abstracts (16.7%), book chapters (2.1%), and conference papers (2.1%) (Figure 2).

**Figure 3:**
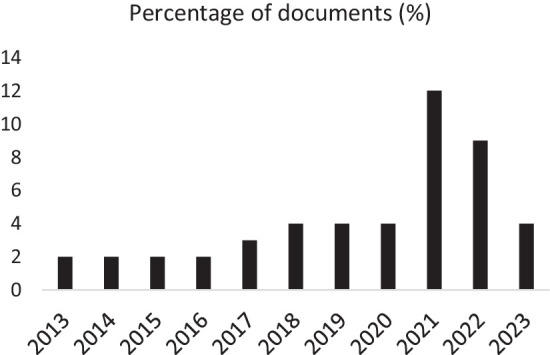
Percentage of documents used from 2013 to 2023.

Regarding the year of publication, the number of documents used for this review was approximately 4% from 2013 to 2020. However, the highest number was obtained in 2021 (27.3%) and dropped 2 years later. This increasing trend can be explained by the abundance of publications on this subject, which is becoming more and more important and a topical issue for decision-makers in order to improve the livestock sector (Figure 3).

### 3.2 Geographical distribution of articles

**Figure 4:**
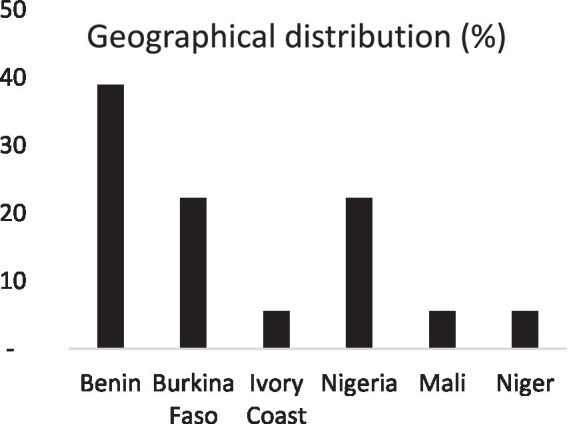
Geographical distribution of articles.

Benin is in first place, with 39% of publications, followed by Burkina Faso and Nigeria (22%) (Figure 4). Ivory Coast, Mali and Niger are included in the same proportion (6%).

### 3.3 Alternative feeds identified

**Figure 5:**
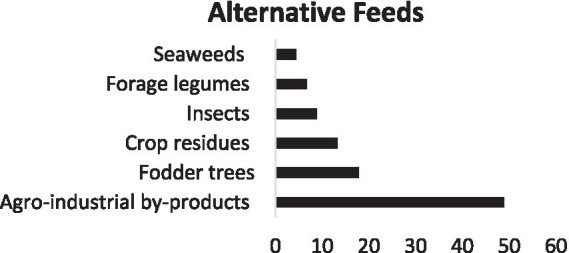
Alternative feeds.

We identified six groups of feeds, distributed as follows: Agro-industrial by-products (48.89%), fodder trees (17.78%), crop residues (13.33%), and insects (8.89%). In addition, we found fodder legumes (6.67%), followed by seaweeds (4.44%) (Figure 5).

### 3.4 Alternative feeds in farming systems

**Figure 6:**
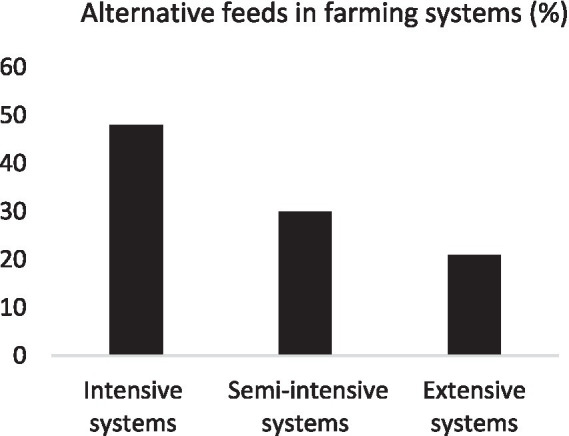
Trend toward alternative feeds in farming systems.

The use of alternative feeds varies by farming system. Our study showed that intensive systems used 48% of the identified feeds, compared with 30% in semi-intensive systems and 21% in extensive systems (Figure 6). The percentage obtained in the intensive systems can be explained by the strong integration of livestock and crops production systems. The low level observed in extensive systems can be explained by a lack of interest in animal feed, followed by a lack of financial resources for the supply of agro-industrial by-products. Difficult access to feed is also due to farmers’ mobility, which takes them far from feed sales outlets. In semi-intensive systems, livestock farming is sometimes seen as a secondary activity, so small herds are common. These farmers are generally located in urban areas, which justifies their limited use of feed.

### 3.5 Factors limiting the availability of alternative feeds

**Figure 7:**
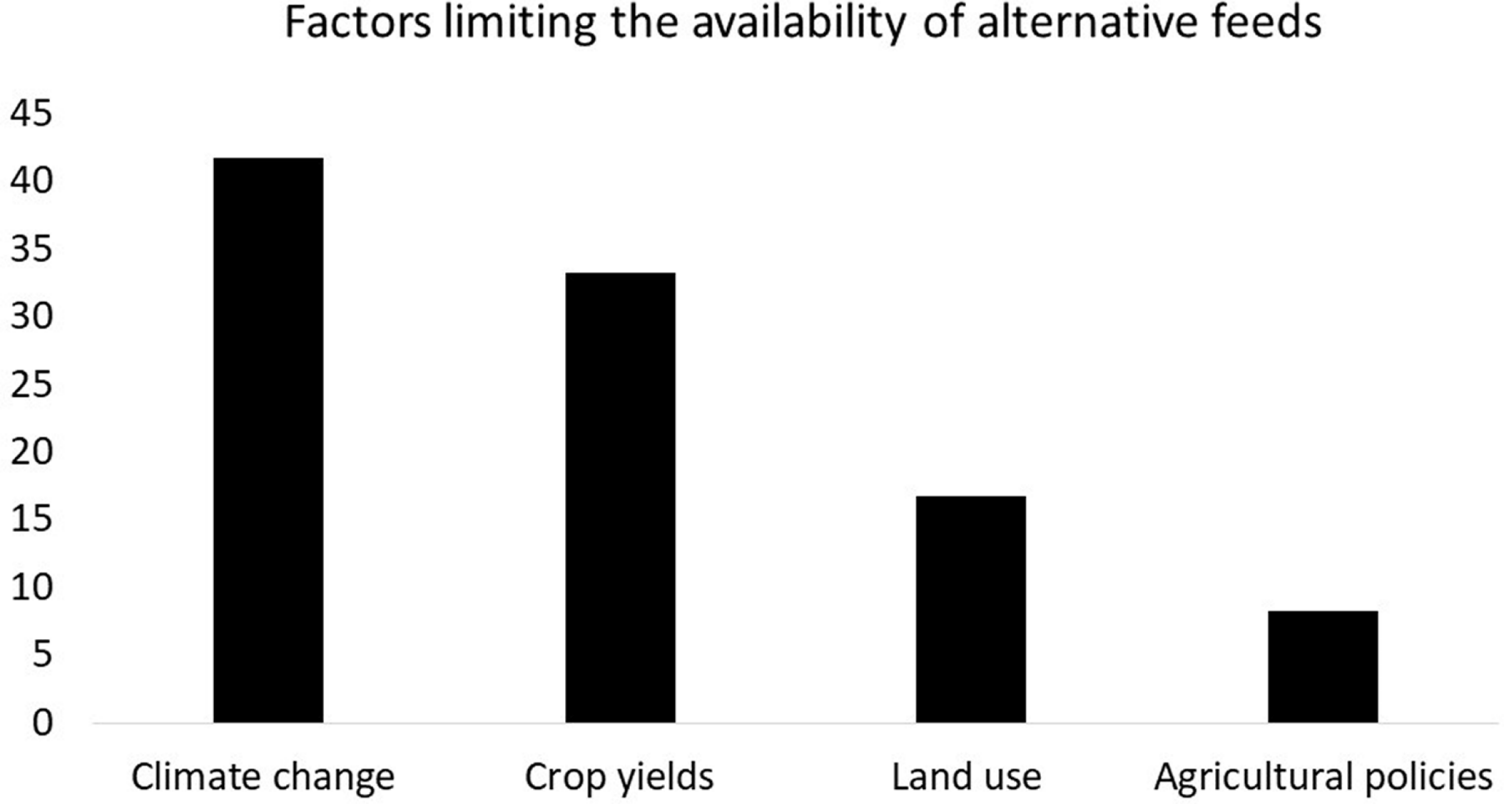
Factors limiting the availability of alternative feeds.

Studies carried out between 2013 and December 2023 (Figure 7) show that several factors limited the availability of alternative feeds. These include climate change (41.8%), which affects not only agricultural land but also crop yields. The latter contributes to animal feed (33.3%). Variations in rainfall, extreme temperatures and the proliferation of harmful insects and pests are all threats to crop yields. In addition, land use (16.8%) due to increasing urbanization limits the production of livestock feed. Agricultural policies (8.3%), with their lack of interest in crop diversification, influence the availability and price of raw materials.

Climate change is expected to have several impacts on feed crops, thereby negatively affecting the availability of alternative feeds while altering their nutritional value and yield at the same time. According to Getu (28), one of the most important effects of climate change on livestock production is the change in feed resources because indirect effects on feed resources can have a significant impact on livestock production, the buffering capacity of ecosystems and their sustainability, the prices of stover and grain, trade-in feeds and changes in feeding options. Drought-induced by climate change and other anthropogenic activities can have adverse effects on horticultural crop production (29). It tends to induce the sprouting of tubers in potatoes (30). The consequences are a reduction in yield, water content and quality of vegetables, such as spinach (30). Drought increases the salinity in the soil, thus affecting osmotic potential and therefore water loss from plant cells, leading to reduced productivity (31). In addition to their basic nutritional value, fruits and vegetables are also rich in biologically active components, such as ascorbic acid, sugars, and phenolics, which provide health benefits (32). The concentration of many biologically active components can increase with increasing CO_2_ levels, but there is also a decrease in protein and mineral content (33). Frequent exposure of fruit to high temperatures (35–40°C) can result in sunburn and loss of texture (34). Since temperature also has an effect on photosynthesis, the impact can be seen in different physio-chemical alterations in fruit and vegetable products, such as sugars, organic acids, firmness, and antioxidant activity (30, 34). In addition, some fruits stored at 0°C (below the recommended temperature), showed a lower incidence of chilling injury (e.g., color and texture of the fruit) than the same fruit harvested from the shaded parts of the tree (34). Post-harvest temperature management of fruits and vegetables is the most important factor in preserving vitamin C. Its losses are accelerated at higher temperatures and with longer storage times (32). Furthermore, in warmer climates, fruits and vegetables are harvested at higher pulp temperatures, which require more energy and refrigerants for proper cooling and may increase product prices (34). Many studies have shown that climate change has a negative impact on seaweeds. According to Khan et al. (35) an increase in greenhouse gas emissions has led to an increase in global average air and ocean temperatures. Increased sea surface temperatures may cause changes in the distribution of seaweed species, particularly those close to their thermal tolerance limits. Moreover, Sunny (36) found that the survival, growth, and reproduction of seaweeds vary with numerous environmental variables, including temperature, nutrient supply via upwelling and runoff, pH, and carbon dioxide concentration itself. As sea levels rise, the distribution and abundance of seagrass and seaweed habitats in any given location decrease. An increase or decrease in salinity impacts seagrass and seaweed. The potential effects of increasing CO_2_ and the impacts of greater UV-B radiation will alter the photosynthesis and productivity of seagrass and seaweed, and the author concluded that there is a critical need for research on the direct effects of the various aspects of global climate change on seaweed and seagrass (36).

### 3.6 Nutritional value of alternative feeds

Alternative feeds are important sources of energy and protein that can be used in ruminant diets because they offer great advantages, such as sustainability, local availability, and resilience to climate change. They include crop residues and agro-industrial by-products, fodder legumes, fodder trees, insects, and algae. Their nutritional value includes dry matter (DM), organic matter (OM), nitrogen, crude cellulose, and fat content. The main methods for determining nutrients are spectrometers, official methods approved by the Association of Official Analytical Chemists, followed by linear regression to determine nutritional values. The nutrient values of alternative feeds are shown in the Table 1.

**Table 1:** Chemical composition of alternative feeds.

Alternative feeds	DM	Ash	OM	CP	NDF	ADF	*CF*	Fat	References
**Agro-industrial by-products**									
*Oil cakes*									
Cottonseed meal	92	6.4	93.5	41.7			11.3	1.8	(37)
	89.86	7.15	92.85	39.02			11.92	3.07	(38)
Palm kernel cake	92.86	2.90	97.1	8.69			11.38	52.40	(39)
Soybean meal	90			45.5				9.1	(40)
Groundnut meal	92	7.5	92.5	34.17			5	10	(41)
Cotton seeds		5.85	94.15	24.34			18.71	24.81	(42)
	91.5			25.5			25.3		(16)
*Roots and tubers by-products*									
Yam peels	89	5.5	94	8	75.3	66.2	4.4	0.3	(43)
	95.3	9.8	90.2	4.9			12.2	3.3	(44)
Cassava peels	92.8	5.5	94.7	5.3	60.9	49.4	10	1.8	(43)
		0.65	99.35	6.24			13.34	1.45	(45)
*Fruit by-products*									
Cashew apples	8.4	0.2	99.8						(46)
Banana peels		15.30	84.7	4.77				13.15	(47)
Mango peels	78.7			3.4	44.7	17.1	8.3	0.4	(48)
Pineapple Pomaces	20.98	2.78	97.22	8.81	21.53	9.75		0.30	(49)
Apple pomaces	92.1	38.4	61.6	7.3		48		4	(50)
Orange peels	92.9	9.8	90.4	6.9	63.2	41.2	14	2.9	(43)
	26.6	3.8	96.2	3.5	10	7.6		1.7	(51)
Pineapple peels	15.08	4.93	95.07	8.01	26.48	11.97		0.35	(49)
		4.4	95.6	5.1			14.8	5.3	(52)
Pineapple crowns	20.62	4.81	95.19	8.48	39.01	20.31		0.68	(49)
Pineapple bud ends	17.07	4.59	95.41	6.54	33.27	15.19		0.37	(49)
Pineapple cores	15.29	1.58	98.42	5.18	10.80	6.85		0.19	(49)
*Cereal by-products*									
Maize bran	92.8	5.1	94.9	14.8	65.6	62.6	6.7	1.6	(43)
	96			12	43	40	35	12	(48)
Sorghum bran	96.16	23.75	76.25	19.54					(17)
Rice bran	93.4	21.8	71.5	3	47.1	11.7	11.4	24.4	(53)
		8–12	88–92	11–16				12–20	(54)
*Legume and pulse by-products*									
Soybean bran		4.5	95.5	18.4			19.7	8.8	(55)
Okara dried soya pulp	93.8	3.9	96.1	34.5			15	11.3	(56)
*Crop residues*									
Banana leaves	33.95	20.52	79.48	11.52			27.73	4.15	(57)
		10.37	89.63	14.98				21	(47)
Rice straw	95.81	13.66	82.12	19.57			27.03	5.2	(58)
	95.7	15	80.6	2.3	87.9	33.7	30.5		(53)
Cowpea hay	92	11.7	88.5	13.5	71.4	56.3	30.5	2.2	(43)
	87.4	11.3	89	16.6	46.4				(59)
Groundnut hay	86.9	6.6	93.5	15.5	65.6	43.5	22.1	10.5	(43)
	94.5	8.12	91.88	17.32			29.75	2.11	(60)
Soybean hay	93.45	6.36	93.64	8.04			42.28	0.49	(61)
Cassava leaves		4.93	95.7	28.73			14.72	9.10	(62)
	27.8	10.4	89.6	25.90	58.2	25.6			(63)
*Fodder legumes*									
Clover		10.1	89.9	18.7			17.1		(64)
Alfalfa	91	11.2	88.8	58.64	10.8	1.2	0.6	11.4	(65)
Lotier				19.71	38.28	28.42	26.35		(66)
				19.5	38.88	28.99	24.39		(67)
*Fodder trees*									
*Leucaena leucocephala*		16.3	83.7	26.5	50.06	23.6			(15)
*Gliricidia sepium*	31.2	9.3	90.7	25.1			13.5		(11)
*Calliandra calothyrsus*		14.3	85.7	21.1	54.9	34.7			(15)
*Faidherbia albida*	90	3.8	96.2	11.4	41.8	30.7			(68)
*Cajanus cajan*	93.68	20.6	79.4	31.99			21.82	13	(69)
	89	8.87	91.13	17.5	46.66	33.33			(70)
*Moringa oleifera*	33	11.5	88.5	25.1	21.9			5.4	(71)
*Lablab purpureus*	89	11.11	88.89	19.23	40	24.44			(70)
*Cassia tora* hay	94.4	17	83	18.81			29.34	2.81	(60)
*Insects*									
Black soldier fly (larvae)		7.1	92.9	40.4			9.7	33.5	(72)
Termites		4.7	95.3	17.2			5.3	3.7	(73)
Housefly maggots	92.7	6.25	93.75	47.6			7.5	25.3	(74)
	93.17	7.86	92.14	56.42			7.14	20.03	(75)
Locusts		2.9	97.1	51.9			13.7	23.1	(76)
*Seaweed*									
Duckweed	5.6	15.9	84.1	29.1	40.1			6.1	(71)
*Asparagopsis taxiformis*		58.3	41.7	13.3	22	9.2		2.3	(18)

DM, dry matter; OM, organic matter; CP, crude protein; CF, crude fiber; NDF, neutral detergent fiber; ADF, acid detergent fiber.

### 3.7 Effect of using alternative feeds on dry matter intake and digestibility

Several studies have shown the efficient use of alternative feeds through improved intake and digestibility (Table 2). According to (77), the highest daily feed intake (285.36 g/day), nitrogen intake (21.75 g/day), and nitrogen balance (9.37 g/day and 5.36 BW^0.75^) were obtained using dried plantain and mango peels and concentrates in a ratio of 50:18:32 in the diet of West African dwarf goats. It is concluded that this ratio has the potential to enhance the nutrient utilization and growth performance of West African dwarf goats. Moreover, Abebe and Tamir (70) found that supplementation with forage legumes led to an increase in total dry matter, crude protein, and NDF intake and suggested that supplementation with *Lablab purpureus* resulted in improved nutrient intake in Wollo Tumele lambs. The authors also found that supplementation of 243 g of pigeon pea or 260 g of cowpea with 200 g of wheat bran per day and per animal could be recommended for intact Wollo Tumele lambs fed grass hay in order to improve feed and/or nutrient intake. Furthermore, Omotoso et al. (78) showed that *Cajanus cajan* had better nutritional values with 15.53% crude protein and 30.55% crude fiber and added that the inclusion of *Cajanus cajan* as a supplement to cassava peels in the goat diet significantly reduced dry matter intake. Crude protein intake increased with increased hay supplementation. Crude protein, crude fiber, NDF, ADF, and ADL intakes were highest in goats fed the diet (25% cassava +75% *Cajanus cajan* hay). Nutrient digestibility and retained nitrogen were highest in the 75% *Cajanus cajan* inclusion treatment. The study found that combining cassava peels with *Cajanus cajan* hay in a 1:3 ratio resulted in an improvement in nitrogen content, digestibility, and optimal levels. According to Kiatti et al. (49), fruit by-products can be used as a base for ruminant feed based on their nutritional content of sugar and fiber. Pineapple by-products, specifically the core and pomace, were found to have low structural carbohydrate content, high *in vitro* degradability, and high volatile fatty acid production. This study suggests that pineapple by-products can be used in ruminant nutrition; the crown, bud end, and peel can be use as fiber sources, while the core and pomace can be used as energy sources.

**Table 2:** Effect of alternative feeds on dry matter intake and digestibility.

Alternative feeds	Replaced feeds	Sup	Inclusion levels	Animal type	DMI	Dig	FCE	References
Dried plantain + mango peels + concentrates	Elephant grass	No	50:18:32	West African Dwarf goats	Inc	Inc	Higher	(77)
Pigeon pea or cowpea or lablab	Grass hay +200 g DM wheat bran	Yes	243, 260, or 225 g DM	Wollo sheep (lambs)	Inc	Inc	Higher	(70)
*Cajanus cajan* hay	Cassava peels	Yes	25:75%	West African Dwarf goats	Dec	Inc	NI	(78)
*Moringa oleifera* stems	Concentrated feed mix	No	25%	Growing lambs	Dec	Dec	Higher	(79)
*Gliricidia sepium*	Cottonseed	No	770:300 (g)	Djallonke sheep	Inc	NI	Higher	(11)
*Leucaena leucocephala*	Cottonseed	Yes	650:300 (g)	Djallonke sheep	Inc	NI	Higher	(11)
Dried banana peels		Yes	20–40%	Djallonke sheep	Inc	Inc	Higher	(12)
Dried cassava		Yes	40%	Djallonke sheep	Inc	Inc	Higher	(12)
*Faidherbia albida* pods		Yes	2 kg	Bull calves	Inc	NI	NI	(13)
*Cassia tora* leaves	Groundnut hay	No	25–50%	Male Djallonke sheep	Inc	Inc	Higher	(60)
Moringa residues (*Moringa oleifera* Lam.)		Yes	22–33%	Young male sheep	Inc	NI	NI	(80)
*Spondias mombin*	*Vitellaria paradoxa*	No		Lactating ewes	Inc	NI	Higher	(14)
Cowpea forage		Yes	18, 75–25%	Dairy cows	NI	NI	NI	(16)
Leaf’s powder of *Spondias mombin* L.		Yes		West African Dwarf sheep	NI	NI	NI	(81)

Sup, supplemented; Inc, increase; Dec, decrease; Dig, digestibility; DMI, dry matter intake; FCE, feed conversion efficiency; NI, no information.

### 3.8 Effects of alternative feeds on average daily gain

Alternative feeds show interesting results in ruminant production, especially for meat production (Table 3). According to Okoruwa et al. (77), dried plantain with mango peels and concentrates in a ratio of 50:18:32 has potential for growth performance in West African dwarf goats, recording a daily weight gain of 33.67 g and 13.98 BW^0.75^. Based on these results, it is therefore concluded that the combination of dried plantain and mango peels in different proportions has good nutritional potential and could be the main component of goat rations. Mahmoud (79) found that *Moringa oleifera* stems are suitable for sheep feed because they can be used without any negative effect on Rahmani lambs. Furthermore, Idrissou et al. (11) studied the fattening performance of Djallonké sheep supplemented with *Gliricidia sepium* and *Leucaena leucocephala* fodder in central Benin. The evolution of the average daily gain per 15-day period was not significantly different between sheep from the first to the third-fortnight experiment. However, in the fourth fortnight, Djallonké sheep supplemented with *Leucaena leucocephala* and *Gliricidia sepium, respectively*, had a significantly higher average daily gain. Abebe and Tamir (70) found that supplementing Wollo Tumele lambs with 243, 260 or 225 g dry matter per animal per day of pigeon pea (*Cajanus cajan*), cowpea (*Vigna unguiculata*) and lablab (*Lablab purpureus*) in addition to *ad libitum* feeding of natural pasture grass hay and 200 g dry matter per animal per day of wheat bran resulted in average daily gains of 34.97, 20.33 and 49.36 respectively. These authors concluded that these forage legumes can be used to improve feed and/or nutrient intake and carcass yield characteristics. Studies on the effects of cassava and banana peels (12) showed that incorporating dried banana peels in the feed at 20% accelerated sheep growth and their average daily weight gain. Moreover, Kiéma et al. (13) showed that the use of *Faidherbia albida* pods improved the average daily gain from the eighth week and, therefore, the growth performance of young bulls. Similarly, studies by (60) on the effects of *Cassia tora* hay on the zootechnical performance of fattening Djallonké sheep fed rations R1 (75% cottonseed cake and 25% *Cassia tora* hay) and R2 (50% cottonseed cake and 50% *Cassia tora* hay) showed that the average daily gain induced by rations R1 and R2 was 80.7 and 71.1 g/animal/day, respectively. Their findings show that using *Cassia tora* leaves as a protein source replacement can improve feed intake, feed efficiency and feed conversion. A study on the combination of *Moringa oleifera* Lam. residues, sorghum stalks and wheat bran (80) showed that dried *Moringa oleifera* residues have the same crude protein content as groundnut hulls or cowpea leaves and total live weight gain varied from 1.88 to 2.35 kg and average daily weight gain from 20.83 to 26.11 g. They concluded that, in the future, livestock farmers will be able to collect, dry and store Moringa residues and use them as fodder in the same way as groundnut hulls or cowpea leaves.

**Table 3:** Effects of alternative feeds on average daily gain.

Alternative feeds	Replaced feeds	Sup	Inclusion levels	Animal type	EMP	ADG (g)	References
Dried plantain + mango peels + concentrates	Elephant grass	No	50:18:32	West African Dwarf goats	Inc	33.67	(77)
Pigeon pea (*Cajanus cajan*)	Grass hay +200 g wheat bran	Yes	243 g	Wollo sheep	Inc	34.97	(70)
Cowpea (*Vigna unguiculata*)	Grass hay +200 g wheat bran	Yes	260 g	Wollo sheep	Inc	20.33	(70)
Lablab (*Lablab purpureus*)	Grass hay +200 g wheat bran	Yes	225 g	Wollo sheep	Inc	49.36	(70)
*Cajanus cajan* hay	Cassava peels	Yes	25:75	West African Dwarf goats	Inc	16.98	(78)
*Moringa oleifera* stems + clover hay	Concentrate feed mixture	No	25	Growing lambs	Inc	223.8	(79)
*Gliricidia sepium*	*Panicum maximum* C1 (*ad-libitum*)	Yes	770 g	Djallonke Sheep	Inc	65.27	(11)
Cotton seed	*Panicum maximum* C1 (*ad-libitum*)	Yes	300 g	Djallonke Sheep	Inc	60.02	(11)
*Leucaena leucocephala*	*Panicum maximum* C1 (*ad-libitum*)	Yes	650 g	Djallonke Sheep	Inc	70.83	(11)
Dried banana peels		Yes	40%	Djallonke Sheep	Inc	48.4	(12)
Dried cassava		Yes	40%	Djallonke Sheep	Inc	51.02	(12)
*Faidherbia albida* pods		Yes	2 kg	Bull calves	Inc	623.02	(13)
*Cassia tora* leaves	Groundnut hay	No	25%	Male Djallonke sheep	Inc	80.7	(60)
Moringa residues (*Moringa oleifera* Lam.)		Yes	22–33%	Young male sheep	Inc	24.8–26.1	(80)
*Spondias mombin*	*Vitellaria paradoxa*	No		Lactating ewes	Inc	88.58	(14)
Leaf’s powder of *Spondias mombin* L. + Galactin		No		West African Dwarf sheep	Inc	99.07	(81)

Sup, supplemented; ADG, average daily gain; Inc, increase; EMP, effet on meat production.

### 3.9 Effects of alternative feeds milk production and composition in ruminants

Results of alternative feeds on milk production show that *Spondias mombin* extract improves milk production in ewes (14) with an average of 100.63 g/day (Table 4). According to Pamo et al. (15) who carried out studies on the chemical composition and effect of supplementation with *Calliandra calothyrsus* and *Leucaena leucocephala* on milk production in goats, fresh leaves of *Calliandra calothyrsus* and *Leucaena leucocephala* doubled production (361 g/d) in dairy goats. Studies by Sanogo et al. (16) on the effect of cereal straw, cowpea fodder, and cotton cake on improving milk production showed that the average cumulative milk production was 140 liters for cows in stalls. Further work like the study by Gbenou et al. (17) on the effect of sorghum meal supplementation on the milk production of cows (Gir × Borgou) showed the production of 3.3 kg milk/day with the supplementation of 2 kg of sorghum meal. Finally, Akouedegni et al. (81) showed that the average daily increase in milk production was 9.92, 14.25, and 18.88% in the single-dose, double-dose, and Galactin groups, respectively, compared to the control group in ewes treated with *Spondias mombin* leaf powder. Similarly, the findings of Seidou et al. (82) showed that forage legumes improve milk production in local cows, even in the dry season in the drylands of Benin.

**Table 4:** Effect of alternative feeds on milk production and composition in ruminants.

Alternative feeds	Replaced feeds	Sup	Inclusion levels	Animal type	EMY	Reference
Dried plantain + mango peels + concentrates	Elephant grass	No	50:18:32	West African Dwarf goats	Inc	(77)
Pigeon pea or cowpea or lablab	Grass hay +200 g DM wheat bran	Yes	243, 260 or 225 g DM	Wollo sheep (lambs)	Inc	(70)
*Spondias mombin*	*Vitellaria paradoxa*	No		Lactating ewes	Inc	(14)
Cowpea forage		Yes	18, 75–25%	Dairy cows	Inc	(16)
Leaf’s powder of *Spondias mombin* L.		Yes		West African Dwarf sheep	Inc	(81)

Sup, supplemented; Inc, increase; EMY, effet on milk yield.

### 3.10 Factors limiting the use of alternative feeds

The first factor limiting the widespread use of alternative feeds in animal nutrition is their high water content, which can often exceed 60–80% making them difficult to handle and store and leading to spoilage (21). Similarly, the seasonal availability of fruits and vegetables and their by-products also has an impact on feed production. In addition, anti-nutritional factors limit their incorporation into the diet. For example, red sorghum is rich in tannin and groundnut, and cotton and palm kernel cake contain aflatoxin, gossypol, and cellulose, respectively. Other factors such as rising food prices and stockouts are major obstacles to their use. Land use, climate change, followed by declining crop yields, and a lack of agricultural policies also affect the availability of alternative feeds. The effects of climate change (83) lead to the reduction of feed and water resources. This is also confirmed by Far (84), who showed that all the effects of climate change lead to local resource reduction due to higher temperatures, CO_2_ affecting grazing land (85). In addition, Abdou et al. (86) showed that climate change causing higher temperatures and long periods of drought thus affecting alternative feeds production. These factors have negative impacts on ruminant farming because alternative feeds became scarce.

### 3.11 Protecting the environment through the use of alternative feeds

To mitigate the effects of climate change and improve agricultural production, the use of alternative feeds is proving to be a better option. Ahmed et al. (18) showed that dietary supplementation of animal fodder with *Asparagopsis taxiformis* at 1 and 2.5% reduced methane production by 21 and 80% respectively, while the inclusion of *Euglena gracilis* in the diet at 10 and 25% reduced methane production by 4 and 11%, respectively, with no negative effect on fermentation parameters. Similarly, *Gryllus bimaculatus* and *Bombyx mori* reduced methane production by 18 and 16%, respectively, (87). In addition, phytochemicals in the feed, such as tannins, saponins, and essential oils, could potentially modify the microbial flora and thereby reduce methanogens in the rumen (88). Moreover, tannins and other phytochemicals containing phenolic groups may be better at binding proteins and slowing down their degradation by rumen microbes (89). Tannins and rumen bacteria interactions or their suppression of fiber digestion may be directly or indirectly beneficial to reducing CH_4_ production (90). According to Moate et al. (91), the addition of polyphenolic compounds to citrus by-products reduced CH_4_ emissions by inhibiting the growth and activity of methanogens such as Methanomicrobium and Methanobrevibacter. The authors also showed that alfalfa hay reduced CH_4_ emissions by 22.6%, with positive stimulation of rumen bacteria and archaea populations. Finally, studies on the chemical and nutritional characteristics of *Cannabis sativa L*. co-products (92), showed that all hemp co-products presented interesting nutritional characteristics, such as a crude protein content always higher than 20% on a dry matter basis, and a high neutral detergent fiber concentration partially lignified. Preliminary results underline that the use of hemp processing residues could be a valid nutrient resource in ruminant diets. The low methane values suggest that these residues could be used in ruminant diets to limit gas emissions, and therefore environmental impact.

### 3.12 Technical options for improving the use of alternative feeds

To ensure the long-term use of alternative feeds, it is important to adopt low-cost preservation methods, such as dehydration and ensiling. These methods can help preserve feeds and improve their use for everytime. Glocusinolates can be removed from soybeans by cooking or toasting. Groundnut cake is detoxified using an aqueous ammonia solution, and *gossypol* is eliminated from cotton cake using iron sulfate (93). In addition, the following incorporation limits must be respected: red sorghum (30%), groundnut cake (25%), cottonseed cake (10%), and palm kernel cake (20%) for more efficient use (43). Furthermore, widespread dissemination of good pre- and post-harvest practices in the mango and cashew nut sectors, for example, must be known and mastered by users to minimize post-harvest losses. It can also ensure the quality of foodstuffs when fruits are harvested at the right stage of ripeness and then processed using an appropriate production process. To improve fruit and vegetable by-products, there is a need to build the capacity of actors in fruit processing, followed by material and financial support.

### 3.13 Prospects for research on alternative feeds

Further research is needed to evaluate the palatability and determine the optimal levels of inclusion of these feeds and by-products, which will be obtained from different plants grown under different conditions and extracted and processed by different methods. Biotechnology will be used to develop fruit varieties that are suitable for animal feed. Similarly, analytical methods must be developed to complete the measurement and categorization of micronutrients and phytochemicals in alternative feeds. In addition, the bioactivity, bioavailability, toxicity, and interactions of phytochemicals in alternative feeds with other feed components should be the subject of future research through *in vitro* and *in vivo* experiments.

### 3.14 Limitations of the review

This study excluded publications prior to 2013, given the evolution of scientific progress and the reliability of results using modern tools. Nevertheless, non-scientific or non-English scientific sources in other fields may contain information not considered in this review. If necessary, other reviews could focus on these sources. This confirms the need to focus attention on alternative feeds and study chemical compounds that limit their use in ruminant feeding. It will also be useful to develop feeding schedules that take into account West African realities to help improve feeding systems.

## 4 Conclusion

This review identified 45 alternative feeds that could benefit the livestock sector but poor management practices lead to livestock underfeeding during the dry season. We need to look at these resources to improve their use. Alternative feeds will be profitable for livestock by improving the processing systems from which they are derived. Based on the diversity of agroecological zones in West Africa and the uneven distribution of livestock, this offers opportunities for the emergence of feed production and marketing. This will provide support for mechanisms to establish and then strengthen feed assessments for livestock in West Africa. It will help to promote and improve livestock resilience to climate change and support policies for sustainable production.

## Author contributions

NA: Conceptualization, Investigation, Methodology, Software, Writing – original draft, Writing – review & editing. AA: Conceptualization, Funding acquisition, Investigation, Methodology, Software, Supervision, Validation, Visualization, Writing – review & editing. HS: Conceptualization, Investigation, Software, Supervision, Writing – review & editing. NB: Methodology, Supervision, Validation, Writing – review & editing. IT: Supervision, Validation, Visualization, Writing – review & editing.

## References

[1] Kassa SK Salifou CFA Dayo GK Ahounou GS Dotché OI Issifou TM. Production de lait des vaches Bororo blanches et Borgou en élevage traditionnel au Bénin. Development. (2016) 28:9.

[2] Nana PD Andrieu N Zerbo I Ouédraogo Y Le Gal P-Y. Agriculture de conservation et performances des exploitations agricoles en Afrique de l’Ouest. Cahiers Agric. (2015) 24:113–22. doi: 10.1684/agr.2015.0743

[3] Musco N Koura IB Tudisco R Awadjihè G Adjolohoun S Cutrignelli MI. Nutritional characteristics of forage grown in south of Benin. Asian Australas J Anim Sci. (2016) 29:51–61. doi: 10.5713/ajas.15.0200 26732328 PMC4698689

[4] Gerber PJ Steinfeld H Henderson B Mottet A Opio C Dijkman J. Tackling Climate Change Through Livestock: A Global Assessment of Emissions and Mitigation Opportunities. Rome: Food and Agriculture Organization of the United Nations (FAO) (2013).

[5] Ha-Duong M., (2013). Les Rapports GIEC, Corpus de la Science du Risque climat? Centre Internationnal de Recherche sur l’Environnement et le Développement, (CIRED), UMR CNRS/Ponts Paris Tech. Available at: https://enpc.hal.science/hal-00846704 (Accessed November 28, 2023).

[6] Harpold AA Kaplan ML Klos PZ Link T McNamara JP Rajagopal S. Rain or snow: hydrologic processes, observations, prediction, and research needs. Hydrol Earth Syst Sci. (2017) 21:1–22. doi: 10.5194/hess-21-1-2017

[7] Bergeret P Maria F. L’agriculture familiale pour valoriser les savoirs et les ressources humaines In: Lacirignola C Graziano da Silva J, editors. Mediterra 2016: Zéro Gaspillage en Méditerranée. Ressources Naturelles, Alimentations et Connaissances, vol. 15. Paris, France: Presses de Sciences Po (2016). 373–84.

[8] Sutton MA Bleeker A Howard CM Erisman JW Abrol YP Bekunda M. Our Nutrient World. The Challenge to Produce More Food and Energy with Less Pollution. UK: Centre for Ecology & Hydrology (2013).

[9] Uwizeye A de Boer IJ Opio CI Schulte RP Falcucci A Tempio G. Nitrogen emissions along global livestock supply chains. Nat Food. (2020) 1:437–46. doi: 10.1038/s43016-020-0113-y

[10] Bakel M. Dardabou O., (2019). Amélioration de la Production Laitière a Base Naturelle (PhD Thesis). Université IBN Khaldoun TIARET.

[11] Idrissou Y Assani SA Alkoiret IT Mensah GA. Performance d’embouche des ovins Djallonké complémentés avec les fourrages de Gliricidia sepium et de Leucaena leucocephala au Centre du Bénin. Bull Rech. Agronomique du Bénin. (2017) 81:1–7.

[12] Montcho M Babatounde S Aboh BA Bougouma-Yameogo V Chrysostome A Mensah GA. Utilisation des sous-produits agricoles et agro-industriels dans l’alimentation des ovins Djallonké au Bénin: perception des éleveurs, préférences et performances de croissance. Afr Sci. (2017) 13:174–87.

[13] Kiéma S Louis K Ouédraogo S Kabor CY. Effet de l’utilisation des gousses de Faidherbia albida sur les performances de croissance des taurillons à l’Ouest du Burkina Faso. Sci Naturelles et Appliquées. (2019) 38:159–68.

[14] Akouedegni CG Koudande OD Ahoussi E Hounzangbe-Adote MS. Effects of leaves extract from *Spondias mombin* L. and *Vitellaria paradoxa* Gaertn F. On west African dwarf (WAD) sheep performance in Republic of Benin. J Anim Sci Adv. (2013) 3:74–82. doi: 10.5455/jasa.20130219104112

[15] Pamo ET Boukila B Fonteh FA Tendonkeng F Kana JR. Composition chimique et effet de la supplémentation avec Calliandra calothyrsus et *Leucaena leucocephala* sur la production laitière et la croissance des chevreaux nains de Guinée. Livest Res Rural Dev. (2005) 17. Available at: http://www.lrrd.org/lrrd17/3/tedo17030.htm (Accessed October 22, 2023).

[16] Sanogo OM Doumbia S Descheemaeker K. Complémentation des bovins laitiers pour l’amélioration de la production de lait et du fumier en milieu paysan dans le cercle de Koutiala. Revue Malienne de Science et de Technologie. (2019):134–43.

[17] Gbenou GX Soule AH Akpo Y Djenontin AJP Imorou HS Babatounde S. Performances d’engraissement et économique des taurillons métis (Gir x Borgou) complémentés avec la drêche sèche de sorgho au pâturage à *Panicum maximum* C1 dans le Nord-Bénin. Afr. Sci. (2020) 17:18–28.

[18] Ahmed E Suzuki K Nishida T. Micro-and macro-algae combination as a novel alternative ruminant feed with methane-mitigation potential. Animals. (2023) 13:796. doi: 10.3390/ani13050796, PMID: 36899652 PMC10000192

[19] Tayengwa T Mapiye C. Citrus and winery wastes: promising dietary supplements for sustainable ruminant animal nutrition, health, production, and meat quality. Sustain For. (2018) 10:3718. doi: 10.3390/su10103718

[20] Mahmoud AG Mohamed M Mahmoud M. Effect of tomato pomace, Citrus and beet pulp on productive performance and Milk quality of Egyptian buffaloes. Pak J Biol Sci. (2020) 23:1210–9. doi: 10.3923/pjbs.2020.1210.1219, PMID: 32981252

[21] Jalal H Giammarco M Lanzoni L Akram MZ Mammi LM Vignola G. Potential of fruits and vegetable by-products as an alternative feed source for sustainable ruminant nutrition and production: a review. Agriculture. (2023) 13:286. doi: 10.3390/agriculture13020286

[22] Devi S Gupta C Jat SL Parmar MS. Crop residue recycling for economic and environmental sustainability: the case of India. Open Agric. (2017) 2:486–94. doi: 10.1515/opag-2017-0053

[23] Salami SA Luciano G O’Grady MN Biondi L Newbold CJ Kerry JP. Sustainability of feeding plant by-products: a review of the implications for ruminant meat production. Anim Feed Sci Technol. (2019) 251:37–55. doi: 10.1016/j.anifeedsci.2019.02.006

[24] García-Rodríguez J Ranilla MJ France J Alaiz-Moretón H Carro MD López S. Chemical composition, in vitro digestibility and rumen fermentation kinetics of agro-industrial by-products. Animals. (2019) 9:861. doi: 10.3390/ani9110861, PMID: 31653022 PMC6912480

[25] Liberati A. The PRISMA statement for reporting systematic reviews and Meta-analyses of studies that evaluate health care interventions: explanation and elaboration. Ann Intern Med. (2009) 151:W. doi: 10.7326/0003-4819-151-4-200908180-00136 19622512

[26] Moher D. Preferred reporting items for systematic reviews and Meta-analyses: the PRISMA statement. Ann Intern Med. (2009) 151:264. doi: 10.7326/0003-4819-151-4-200908180-00135 19622511

[27] Berrang-Ford L Ford JD Paterson J. Are we adapting to climate change? Glob Environ Chang. (2011) 21:25–33. doi: 10.1016/j.gloenvcha.2010.09.012

[28] Getu A. The effects of climate change on livestock production, current situation and future consideration. Int J Agric Sci. (2015) 5:494–9.

[29] Snyder RL. Climate change impacts on water use in horticulture. Horticulturae. (2017) 3:27. doi: 10.3390/horticulturae3020027

[30] Prasad BVG Chakravorty S. Effects of climate change on vegetable cultivation-a review. Nat Environ Pollut Technol. (2015) 14:923.

[31] Myers SS Smith MR Guth S Golden CD Vaitla B Mueller ND. Climate change and global food systems: potential impacts on food security and undernutrition. Annu Rev Public Health. (2017) 38:259–77. doi: 10.1146/annurev-publhealth-031816-044356, PMID: 28125383

[32] Parajuli R Thoma G Matlock MD. Environmental sustainability of fruit and vegetable production supply chains in the face of climate change: a review. Sci Total Environ. (2019) 650:2863–79. doi: 10.1016/j.scitotenv.2018.10.019, PMID: 30373063

[33] Ayyogari K Sidhya P Pandit MK. Impact of climate change on vegetable cultivation-a review. Int. J. Agric. Environ. Biotechnol. (2014) 7:145–55. doi: 10.5958/j.2230-732X.7.1.020

[34] Mattos LM Moretti CL Jan S Sargent SA Lima CEP Fontenelle MR. Climate changes and potential impacts on quality of fruit and vegetable crops In: Emerging Technologies and Management of Crop Stress Tolerance. Cambridge: Academic Press. (2014). 467–86.

[35] Khan AH Levac E Van Guelphen L Pohle G Chmura GL. The effect of global climate change on the future distribution of economically important macroalgae (seaweeds) in the Northwest Atlantic. FACETS. (2018) 3:275–86. doi: 10.1139/facets-2017-0091

[36] Sunny AR. A review on effect of global climate change on seaweed and seagrass. Communities. (2017) 28:8.

[37] Kumar BP Ramudu KR Devi BC. Mini review on incorporation of cotton seed meal, an alternative to fish meal in aquaculture feeds. Int J Biol Res. (2014) 2:99–105. doi: 10.14419/ijbr.v2i2.3274

[38] Thirumalaisamy G Purushothaman MR Kumar PV Selvaraj P Natarajan A Senthilkumar S. Nutritive and feeding value of cottonseed meal in broilers – a review. Adv Anim Vet Sci. (2016) 4:398–404. doi: 10.14737/journal.aavs/2016/4.8.398.404

[39] Okonkwo CO Ozoude UJ. The impact of processing on the nutritional, mineral and vitamin composition of palm kernel nut (*Elaeis guineensis*). Afr J Food Sci. (2015) 9:504–7. doi: 10.5897/AJFS2015.1290

[40] Juin H. Célia B. Dalila F. Antoine R., (2015). Valeur Nutritionnelle de Sources de Protéines Pour L’alimenttation des Volailles en Production Biologique Résultat des Essais de Digestibilités. INRA-EASM-17700 SURGERES. 7p.

[41] Jain S Bhati D Saxena N. Incorporation of groundnut meal in selected products after removal of aflatoxin. J Hum Ecol. (2014) 48:341–6. doi: 10.1080/09709274.2014.11906803

[42] Zubair MF Ibrahim OS Atolani O Hamid AA. Chemical composition and nutritional characterization of cotton seed as potential feed supplement. J Turk Chem Soc Sec A Chem. (2021) 8:977–82. doi: 10.18596/jotcsa.906949

[43] Montcho M Babatoundé S Aboh BA Bahini MJD Chrysostome A Mensah GA. Disponibilité, valeurs marchande et nutritionnelle des sous-produits agricoles et agroindustriels utilisés dans l’alimentation des ruminants au Bénin. Eur Sci J. (2016) 12:422–41. doi: 10.19044/esj.2016.v12n33p422

[44] Omole AJ Okpeze CN Fayenuwo JA Olorungbohunmi TO. Effects of partial replacement of maize with yam peel (Discorea rotundata) in diet of juvenile snails (Archachatina marginata). Afr J Agric Res. (2013) 8:1361–4. doi: 10.5897/AJAR11.1490

[45] Akinyele BJ Olaniyi OO Jeff-Agboola YA. Effect of fermentation on chemical composition of cassava peels. Asian J Plant Sci Res. (2017) 7:31–8.

[46] Hêdiblè LG Adjou ES Tchobo FP Agbangnan P Ahohuendo B Soumanou MM. Caractérisation physico-chimique et morphologique de trois morphotypes de pommes d’anacarde (Anacardium Occidental L.) pour leur utilisation dans la production d’alcool alimentaire et de boissons spiritueuses. J Appl Biosci. (2017) 116:11546–56.

[47] Kumari P Gaur SS Tiwari RK. Banana and its by-products: a comprehensive review on its nutritional composition and pharmacological benefits. eFood. (2023) 4:e110. doi: 10.1002/efd2.110

[48] Kiendrebeogo T Youssouf ML Georges I Chantal-Yvette K-Z. Procédés de production d’aliments non conventionnels pour porcs à base de déchets de mangues et détermination de leurs valeurs alimentaires au Burkina Faso. J Appl Biosci. (2013) 67:5261–70. doi: 10.4314/jab.v67i0.95047

[49] Kiatti DD Vastolo A Koura BI Vitaglione P Cutrignelli MI Calabrò S. The chemical characteristics and in vitro degradability of pineapple by-products as potential feed for ruminants. Animals. (2023) 13:3238. doi: 10.3390/ani13203238 37893963 PMC10603704

[50] Steyn L Meeske R Cruywagen CW. The effect of replacing maize with dried apple pomace in the concentrate on performance of Jersey cows grazing kikuyu pasture. Anim Feed Sci Technol. (2018) 239:85–93. doi: 10.1016/j.anifeedsci.2018.02.012

[51] Castrica M Rebucci R Giromini C Tretola M Cattaneo D Baldi A. Total phenolic content and antioxidant capacity of Agri-food waste and by-products. Ital J Anim Sci. (2019) 18:336–41. doi: 10.1080/1828051X.2018.1529544

[52] Feumba R Rani A Manohar RS. Chemical composition of some selected fruit peels. Eur. J. Food Sci. Technol. (2016) 4:12–21.

[53] Blama Y Ziébé R Zoli AP. Valeur nutritive et efficacité économique des ingrédients alimentaires utilisés dans l’alimentation des ruminants en zone semi-aride du Cameroun. Afr. Sci. (2018) 14:257–73.

[54] Spaggiari M Dall’Asta C Galaverna G Del Castillo Bilbao MD. Rice bran by-product: from valorization strategies to nutritional perspectives. Food Secur. (2021) 10:85. doi: 10.3390/foods10010085, PMID: 33406743 PMC7824317

[55] Elawad RMO Yang TA Ahmed AHR Ishag KEA Mudawi HA Abdelrahim SMK. Chemical composition and functional properties of wheat bread containing wheat and legumes bran. Int. J. Food Sci. Nutr. (2016) 1:10–5.

[56] Alabi CDA Azalou M Adjassin JS Assani AS Worogo HSS Idrissou Y. Effet de la granulation d’un aliment avec pulpe de soja (okara) sur l’engraissement de lapins locaux au Nord-Bénin. Livest. Res. Rural Dev. (2019) 31:11.

[57] Mat K Taufik HA Rusli ND Hasnita CH Al-Amsyar SM Rahman MM. Effects of fermentation on the nutritional composition, mineral content and physical characteristics of banana leaves In: IOP Conference Series: Earth and Environmental Science, vol. 596: IOP Publishing (2020). 012089.

[58] Tendonkeng F Essie FMN Mboko AV Lemoufouet J Miégoué E Zogang BF. Effet du niveau d’incorporation de mélasse et de la source du liquide ruminal sur la digestibilité in vitro de la paille de riz traitée à l’urée. Livest. Res. Rural Dev. (2016) 28.

[59] Corea EE Aguilar JM Alas NP Alas EA Flores JM Broderick GA. Effects of dietary cowpea (*Vigna sinensis*) hay and protein level on milk yield, milk composition, N efficiency and profitability of dairy cows. Anim Feed Sci Technol. (2017) 226:48–55. doi: 10.1016/j.anifeedsci.2017.02.002

[60] Sidibé S Tangara M Cisse SM Doumbia S Maïga AM Mallé B. Effets de la fane de cassia tora sur les performances zootechniques des beliers djallonke en station. Revue Malienne de Science et de Technologie. (2019) 21:16–25.

[61] Adamu HY Adesina OA Abdu SB Hassan MR Dung DD Bawa GS. Comparative study on intake, digestibility and nitrogen balance of cowpea, groundnut and soybean hays in a mixed diet fed red Sokoto bucks. Niger. J. Anim. Sci. (2016) 18:162–72.

[62] Jamil SS Bujang A. Nutrient and antinutrient composition of different variety of cassava (*Manihot esculenta* Crantz) leaves. Jurnal Teknologi. (2016) 78:59–63. doi: 10.11113/jt.v78.9024

[63] Leguizamón AJ Rompato KM Hoyos RE Audisio MC. Nutritional evaluation of three varieties of cassava leaves (*Manihot esculenta* Crantz) grown in Formosa, Argentina. J Food Compos Anal. (2021) 101:103986. doi: 10.1016/j.jfca.2021.103986

[64] Morel I Delagarde R Dufey P-A Schmid E Aragon A Sonet C. Ingestion élevée d’un mélange graminées-trèfles-chicorée par des bovins à l’engrais. Agrarforschung Schweiz-Recherche Agronomique Suisse. (2021) 12:45–56.

[65] Grela ER Pietrzak K. Production technology, chemical composition and use of alfalfa protein-xanthophyll concentrate as dietary supplement. J. Food Process. Technol. (2014) 5:5. doi: 10.4172/2157-7110.1000373

[66] Churkova B Bozhanska T Naydenova Y. Feeding value of bird’s-foot trefoil (*Lotus corniculatus* L.) cultivar under conditions of the central northern part of Bulgaria. Banat’s. J Biotechnol. (2016) VII:38–45. doi: 10.7904/2068-4738-VII(14)-38

[67] Wróbel B Zielewicz W. Nutritional value of red clover (*Trifolium pratense* L.) and birdsfoot trefoil (*Lotus corniculatus* L.) harvested in different maturity stages. J Res Appl Agric Eng. (2019) 64:14–9.

[68] Sana Y Kiéma S Sanou J Kondombo SR Sawadogo L Kabore-Zoungrana C. Comparaison des performances de croissance chez les lapins nourris avec trois types de rations alimentaires à base de panicum maximum C1, de gousses de Faidherbia Albida. Rev Ivoir Sci Technol. (2020) 35:270–87.

[69] Sahu M Vermaand D Harris KK. Phytochemicalanalysis of the leaf, stem and seed extracts of *Cajanus cajan* L (Dicotyledoneae: Fabaceae). World J Pharm Pharm Sci. (2014) 3:694–733.

[70] Abebe H Tamir B. Effects of supplementation with pigeon pea (Cajanus cajun), cowpea (*Vigna unguiculata*) and lablab (*Lablab purpureus*) on feed intake, body weight gain and carcass characteristics in Wollo sheep fed grass hay. Int J Adv Res Biol Sci. (2016) 3:280–95.

[71] Halmemies-Beauchet-Filleau A Rinne M Lamminen M Mapato C Ampapon T Wanapat M. Alternative and novel feeds for ruminants: nutritive value, product quality and environmental aspects. Animal. (2018) 12:s295–309. doi: 10.1017/S1751731118002252, PMID: 30318027

[72] Kierończyk B Rawski M Pawelczyk P Różyńska J Golusik J Mikolajczak Z. Do insects smell attractive to dogs? A comparison of dog reactions to insects and commercial feed aromas-a preliminary study. Ann Anim Sci. (2018) 18:795–800. doi: 10.2478/aoas-2018-0012

[73] Adeyeye EI Olaleye AA. Chemical composition and mineral safety index of five insects commonly eaten in south West Nigeria. FUW Trends Sci Technol J. (2016) 1:139–44.

[74] Saleh HH. Review on using of housefly maggots (*Musca domestica*) in fish diets. J Zool Res. (2020) 2. doi: 10.30564/jzr.v2i4.2190

[75] Wang L Li J Jin JN Zhu F Roffeis M Zhang XZ. A comprehensive evaluation of replacing fishmeal with housefly (*Musca domestica*) maggot meal in the diet of Nile tilapia (*Oreochromis niloticus*): growth performance, flesh quality, innate immunity and water environment. Aquac Nutr. (2017) 23:983–93. doi: 10.1111/anu.12466

[76] Althwab SA Alhomaid RM Ali RF Mohammed El-Anany A Mousa HM. Effect of migratory locust (Locusta migratoria) powder incorporation on nutritional and sensorial properties of wheat flour bread. Br Food J. (2021) 123:3576–91. doi: 10.1108/BFJ-11-2020-1052

[77] Okoruwa MI Adewumi MK Njidda AA. Nutrient utilization and growth performance of west African dwarf goats fed with elephant grass or different proportions of plantain and mango peels. World J Agric Sci. (2013) 1:194–202.

[78] Omotoso OB Noah FA Adebayo AJ. Nitrogen metabolism, digestibility and blood profile of west African dwarf goats fed dietary levels of *Cajanus cajan* as supplement to cassava peels. J Rangeland Sci. (2019) 9:13–23.

[79] Mahmoud AEM. Effect of feeding on *Moringa oleifera* stems on productive performance of growing lambs. Egypt J Nut Feeds. (2013) 16:281–92.

[80] Malam MA Dan AG Issa S Yahoussa G Karimou M Bagnan S. Performance zootechnique des jeunes ovins mâles nourris en complémentation au résidu de moringa (*Moringa oleifera* Lam.) au Niger. Int J Biol Chem Sci. (2021) 15:2050–7. doi: 10.4314/ijbcs.v15i5.28

[81] Akouedegni CG Adenile AD Olounlade PA Ahoussi E Hamidou HT Hounzangbe-Adote MS. Lactogenic effects of the leaf’s powder of *Spondias mombin* L. on west African dwarf (WAD) sheep performance and serum prolactin level in Republic of Benin. Indian J Anim Res. (2020) 54:254–8. doi: 10.18805/ijar.B-842

[82] Assani SA Offoumon OTLF Worogo SHS Houaga I Yarou AK Azalou M. The effect of the silvopastoral system on milk production and reproductive performance of dairy cows and its contribution to adaptation to a changing climate in the drylands of Benin (West-Africa). Front. sustain. food syst. (2023) 7:1–113. doi: 10.3389/fsufs.2023.1236581

[83] Cisse P., (2014). Comportements des Éleveurs Sahéliens Maliens Face à la Variabilité Climatique. Edition Universiaires de Côte d’Ivoire, 107–21.

[84] Far Z., (2016). Les Élevages Bovins de la Région Semi-Aride de Sétif face au Changement Climatique (PhD Thesis). ENSA.

[85] Vigne M Blanfort V Vayssières J Lecomte P Steinmetz P. Contraintes sur l’élevage dans les pays du Sud: les ruminants entre adaptation et atténuation. Changement climatique et agricultures du monde. Collection Agricultures et défis du monde, Cirad-AFD. In: Torquebiau E, editor. Editions Quae. (2015):123–36.

[86] Abdou H Karimou IA Harouna BK Zataou MT. Perception du changement climatique des éleveurs et stratégies d’adaptation aux contraintes environnementales: cas de la commune de Filingué au Niger. Rev Elev Med Vet Pays Trop. (2020) 73:81–90. doi: 10.19182/remvt.31873

[87] Ahmed E Fukuma N Hanada M Nishida T. Insects as novel ruminant feed and a potential mitigation strategy for methane emissions. Animals. (2021) 11:2648. doi: 10.3390/ani11092648 34573617 PMC8471967

[88] Kamra DN Pawar M Singh B. Effect of plant secondary metabolites on rumen methanogens and methane emissions by ruminants. In: Patra A, editor. Diet. Phytochem. Microbes. Dordrecht: Springer. (2012):351–70.

[89] Patra AK. The effect of dietary fats on methane emissions, and its other effects on digestibility, rumen fermentation and lactation performance in cattle: a meta-analysis. Livest Sci. (2013) 155:244–54. doi: 10.1016/j.livsci.2013.05.023

[90] Vasta V Daghio M Alice C Buccioni A Andrea S Viti C. Plant polyphenols and rumen microbiota responsible for fatty acid biohydrogenation, fiber digestion, and methane emission: experimental evidence and methodological approaches. J Dairy Sci. (2019) 102:3781–804. doi: 10.3168/jds.2018-14985, PMID: 30904293

[91] Moate PJ Williams SRO Torok VA Hannah MC Ribaux BE Tavendale MH. Grape marc reduces methane emissions when fed to dairy cows. J Dairy Sci. (2014) 97:5073–87. doi: 10.3168/jds.2013-7588, PMID: 24952778

[92] Vastolo A Calabrò S Pacifico S Koura BI Cutrignelli MI. Chemical and nutritional characteristics of *Cannabis sativa* L. co-products. Anim Physiol Nutr. (2021) 105:1–9. doi: 10.1111/jpn.13557, PMID: 34448247 PMC8518064

[93] Toléba SS Youssao AKI Dahouda M Missainhoun UMA Mensah GA. Identification et valeurs nutritionnelles des aliments utilisés en élevage d’aulacodes (*Thryonomys swinderianus*) dans les villes de Cotonou et Porto-Novo au Bénin. Bulletin de la Recherche Agronomique du Bénin. (2009) 64:1–10.

