# Erratum: Livestock feed resources used as alternatives during feed shortages and their impact on the environment and ruminant performance in West Africa: a systematic review

**DOI:** 10.3389/fvets.2024.1449218

**Published:** 2024-07-01

**Authors:** Frontiers Production Office

**Affiliations:** Frontiers Media SA, Lausanne, Switzerland

**Keywords:** nutritional value, environmental impact, ruminants, alternative feeds, West Africa

Due to a production error, the initials of authors MK, CI, YT and EA were included in the Contributions section, even though they were no longer co-authors on this paper. Their contribution is mentioned in the Acknowledgements section. The corrected contribution section reads as follows:

## Author contributions

NA: Conceptualization, Investigation, Methodology, Software, Writing – original draft, Writing – review & editing. AA: Conceptualization, Funding acquisition, Investigation, Methodology, Software, Supervision, Validation, Visualization, Writing – review & editing. HS: Conceptualization, Investigation, Software, Supervision, Writing – review & editing. NB: Methodology, Supervision, Validation, Writing – review & editing. IT: Supervision, Validation, Visualization, Writing – review & editing.

The corrected Acknowledgements section reads as follows:

